# Floating Kidneys

**Published:** 1882-11

**Authors:** 


### Floating Kidneys.

Skorczewsky, at a watering-place in Galicia, found 32 females and 3 males with floating kidney, out of 1030 females and 392 males examined. In 19 the right organ and in 11 the left was affected. In 5 (pluriparæ) both organs floated. S. thinks the affection far more common than supposed and that physicians overlook its existence. None of the 35 above mentioned were sent on account of the kidneys. The condition depends, he thinks, chiefly upon disappearance of circumrenal fat, atony of tissues from acute fevers, and pressure of hypertrophied abdominal viscera. Co-existence with malarial hypertrophy of spleen was often observed (most commonly in left displacement) and the author strongly recommends examination of kidneys in all malarial affections.—*London Med. Rec.*

